# Growth status of small for gestational age (SGA) Indian children from two socioeconomic strata

**DOI:** 10.1186/1687-9856-2015-S1-P33

**Published:** 2015-04-28

**Authors:** Vaman Khadilkar, Rubina Mandlik, Sonal Palande, Meghna Chawla, Ruchi Nadar, Shashi Chiplonkar, Anuradha Khadilkar

**Affiliations:** 1 Hirabai Cowasji Jehangir Medical Research Institute, Jehangir Hospital, Pune, Maharashtra, India; 2 Smt. Kashibai Navale Medical College and General Hospital, Pune, Maharashtra, India

## Aims

To assess growth and factors associated with growth in children born SGA from two socio economic strata in comparison to age and gender matched healthy controls.

## Methods

Retrospective study conducted at two hospitals in Pune, 0.5 to 5 years old 618 children – 189 SGA from Upper Socio-economic Strata (USS), 217 SGA from Lower Socio-economic Strata (LSS) and 212 appropriate for gestational age (AGA) healthy controls were randomly selected. Birth history, maternal history, socio-economic status, length/height and weight of children were recorded. Anthropometric data were converted to Z scores (HAZ, WAZ) using WHO AnthroPlus software [1]. Data on neonatal morbidity and feeding history of children were recorded (analysis in progress).

## Results

Mean ages of all 3 groups were similar (2.7 years for USS, 2.8 for LSS and 2.9 for controls). The HAZ and WAZ of the SGA group were significantly lower as compared to the controls (p < 0.05), and that of the LSS SGAs were lower than that of the USS SGAs (p<0.05). The percentage of children who were stunted (HAZ < -2.0) were 32% in USS and 49% in LSS (p< 0.05 for all). The percentage of stunted children in the USS SGA group at 2 years was 29% and at 5 years was 17% while in the LSS SGA group, 54% of children were found to be stunted at 2 years and 46% at 5 years. To determine factors associated with stunting in SGA children, generalized linear model (GLM) was used. GLM revealed that a normal vaginal delivery (β = 0.625) and mother’s age (β = 0.072) were positively associated with risk of stunting, high SES (β = -0.830), absence of major illness (β = -1.01), higher birth weight (β = -1.34) were negatively associated (p for all < 0.05).

## Conclusion

Children born SGA showed relatively poor growth as compared to healthy controls. Special attention to growth is necessary especially in children from the lower socio economic strata, very low birth weight babies and those with major illnesses during early years of life.
